# An Answer to Certain Remarks by the Editor of the Medico-Chirurgical Journal, on a Paper on Puerperal Irritability (Published in the Number of This Journal for February)

**Published:** 1825-05

**Authors:** C. Waller


Art. V.—*An Answer to certain Remarks by the Editor of the Medico-Chirurgical Journal, on a Paper on Puerperal Irritability (published in the Number of this Journal for February). By* C. Waller, Esq. 110.

In the last Number of the Medico-Chirurgical Review, (No. 4, New Series, p. 503,) the Editor, in noticing my paper on Puerperal Irritability, published in the February Number of your valuable Journal, has made some observations, to which, in justice to myself, I feel called upon to reply. He begins with the following remark :—" Mr. Waller, the author of this paper, appears to have some *obscure views* of the principles we laid down in the second Number of the present series of our Journal;" and again—" Whether this affection be noticed by authors, Mr. Waller and our readers will best judge by turning to the article in our second Number, to which we have referred them. In point of fact, the cases here adduced are apt confirmations of the *principles* we there laid down." On perusing these observations, it was very natural for me to refer to the

Number of the Review alluded to, with the full expectation of there finding a clear and accurate description of the *symptoms* of this affection, with its appropriate *treatment*. Judge then of my disappointment, when I found the subject merely hinted at, and in the most cursory manner. Thus, for example, (p. 416,) "*All* the symptoms which characterise phrenitis may occur from intestinal irritation, and *some of them* from loss of blood:" what symptoms arise from the one cause, and what from the other, we are not informed.

The article referred to is an analysis of Mr. Hey's work on Puerperal Fever; and, in his remarks on the ninth case detailed in that work, the Editor observes—"This case was one of a mixed character; we are far from regarding it as one of pure peritonitis; we even doubt whether there was any peritoneal inflammation at all: but there can be no doubt that there was intestinal irritation, and *some of the effects* of loss of blood." A little further on, in alluding to the same case,—"We doubt not there was intestinal irritation: we are certain that *some of the symptoms* arose from loss of blood. Whether the affection in the abdomen was inflammation, and what was the *nature* and *cause* of the affection of the head, *we will not pretend to determine.*" Again, in noticing Case 28th, in the Appendix to Mr. Hey's work,—"This case presents *many* of the symptoms of loss of blood." On turning to this case, your readers will find it a very interesting one of uterine inflammation, occurring in a very delicate female after hemorrhage, in which, "notwithstanding the unfavourable state of the patient, we took away twenty-four ounces of blood." The uneasiness of the abdomen yielded entirely, and the head-affection was much relieved after this bleeding; but this latter symptom again returned after the purgative medicines had acted freely. "As the abdomen remained free from pain, and the uterine enlargement was subsiding, we gave," says Mr. Hey, "a table-spoonful of wine in her gruel, on account of the distressing symptoms in the head; which we *now* attributed more to the loss of blood than the fever." I think Dr. Armstrong, whilst on the subject of mania, distinctly alludes to the prejudicial effects which repeated copious venesection produces in the brain; and indeed it is a fact which we must have all witnessed, not only after large bleedings, but after profuse evacuations of any kind.

The Editor of the Medico-Chirurgical Review states that I entertain "obscure views" of the principles he has laid down, and that my cases are apt confirmations of them. Now I would ask, *what* principles has he laid down, and *how* are they to be made use of, either in the discrimination or the treatment of this affection? All that we can learn from him in regard to symptoms, is "that many of the symptoms of phrenitis occur from loss of blood;" and with respect to the treatment, he merely says, when the head is affected, we are to "*suspect* intestinal irritation, and turn our attention to this source." In fact, from the whole tenor of the article alluded to, it is plain that the Editor wishes to impress upon the minds of his readers the urgent necessity of attending to the state of the bowels when the head is affected, (and of these cases I have seen examples in my practice;) but, whenever allusion is made to any other source of cerebral disturbance, it is done in a most vague and unsatisfactory manner.

With regard to the peculiar state which I have endeavoured to describe, I contend (and in so doing I am borne out by facts,) that it does not necessarily or invariably depend upon loss of blood, although this occurrence appears to be a powerfully exciting cause. The fabric of some females is so delicately constituted, their nervous system so easily excited, their state of excitability so heightened by their situation, that the mere act of labour, unaccompanied by any untoward symptom, is sufficient to produce the effect.

The following queries are proposed by the Editor, in concluding his remarks:—"Now, we would ask, if hemorrhage was the cause of this affection, was blood-letting the proper remedy? Is it not plain that the head was relieved only by the substitution, and during the continuance, of the syncope?" I should have thought that a sufficient reply had been given to these questions. I have stated that little or no benefit was derived from remedies of an antiphlogistic nature; but few, I think, would blame me for taking away eight or ten ounces of blood, when the pulse was one hundred and possessed considerable strength, and when there was *severe pain.* In all the other cases I have witnessed, (and in this also, with the exception of that day,) the pain has not been acute, but of a dull and oppressive kind: I mean, of course, when every thing around was quiet. I have no where advocated this practice, for the result showed that venesection was not the proper remedy, although the appearances of the blood presented what are usually considered the characters of active inflammation; for it was cupped and buffed, and the coagulum was exceedingly firm.

"Does not the effect of the opium on the pulse and symptoms, teach us not only what was the nature of the case, but what is the proper remedy in such cases?" Surely, this question is sufficiently answered by the publication of the case. What object can any one have in reporting the treatment of diseases, unless it be that of recommending what he conceives to be the proper treatment?

"We would wish to be informed what *good* could be expected from digitalis?" At our first consultation, there appeared to be so much *power* in the system, as well as frequency of pulse, that Dr. Blundell and myself considered ourselves fully justified in its exhibition. It is a very easy thing, when we know how a case has terminated, and what was the effect of each particular remedy, to ask what good could be expected from this or the other medicine; but, in the present state of our knowledge, it is not always easy to say, before-hand, what those effects will be.

In conclusion, allow me to say that the foregoing observations were not written under the influence of angry feelings toward the Editor in question, but simply to counteract an opinion which the perusal of his remarks is calculated to convey,—viz. that I had been acting upon *his* principles, and had published them to the world as *my own*. I have carefully reperused the article he has referred to, and also my paper, and I do not perceive that I can, with any degree of justice, be charged with so doing.

*Aldersgate-street; April 2,* 1825.

